# JAK Inhibitor and Crohn’s Disease

**DOI:** 10.3390/biomedicines13061325

**Published:** 2025-05-29

**Authors:** Mengyan Xu, Shi Wang, Sanping Xu, Rui Gong

**Affiliations:** 1 Health Management Center, Union Hospital, Tongji Medical College, Huazhong University of Science and Technology, Wuhan 430022, China; xuhoozi@163.com; 2 Department of Breast and Thyroid Surgery, Union Hospital, Tongji Medical College, Huazhong University of Science and Technology, Wuhan 430022, China; wangshi505213@163.com

**Keywords:** Crohn’s disease, JAK inhibitor, upadacitinib, filgotinib

## Abstract

Crohn’s disease is a chronic inflammatory granulomatous disease of the gastrointestinal tract. The global incidence and prevalence of Crohn’s disease have significantly increased, largely due to genetic susceptibility, environmental changes, and advancements in diagnostic technology. In recent years, the pharmacologic treatment of Crohn’s disease has been rapidly changing, and although biologics have improved the prognosis of patients to a certain extent, they still have certain limitations. Oral small molecule drugs like JAK inhibitors have become a research hotspot because of their advantages of targeting and regulating the JAK/STAT pathway, convenient administration, and rapid onset of action. JAK inhibitors exhibit divergent therapeutic profiles. Clinical trials have shown that tofacitinib demonstrates limited efficacy in Crohn’s disease management. Filgotinib initially showed clinical remission in phase 2 trials; while its subsequent phase 3 studies failed to demonstrate consistent endoscopic improvement. In contrast, upadacitinib achieved notable clinical remission rates during both induction and maintenance phases of phase 2 trials. However, long-term safety concerns, including thromboembolic events, cardiovascular events, opportunistic infections, and potential malignancy risks, warrant cautious clinical application. This article systematically reviews the pathophysiology of Crohn’s disease, and the evidence for the efficacy and safety of JAK inhibitors to guide clinical practice and research.

## 1. Introduction

Crohn’s disease (CD) is a chronic inflammatory granulomatous disease of the gastrointestinal tract characterized by segmental, asymmetrical, and transmural inflammation. Lesions can occur anywhere in the gastrointestinal tract, from the oral cavity to the perianal area, and most commonly affect the terminal ileum and nearby colon. Typical clinical manifestations include abdominal pain, diarrhea, and weight loss, often accompanied by extraintestinal complications to joints, skin, eyes, and oral mucosa. The disease is characterized by recurrent episodes alternating with remission, and some patients may require surgery due to complications or continued progression of symptoms even after receiving standardized treatment [1,2].

Epidemiological data show that CD, previously considered to be a highly prevalent disease in Western societies, has shown a trend of globalization and growth in recent years. In developed countries such as North America, the incidence rate ranges from 10 to 20 cases per 100,000 person-years, while the prevalence rate is about 200 to 500 cases per 100,000 people [3]. Notably, in countries like those in Asia, which previously had low incidence and prevalence rates, the annual incidence of Crohn’s disease has increased to 0.54 cases per 100,000 people, along with a rise in prevalence [4]. With regard to genetic factors, mutations in genes such as NOD2 and IL23R can lead to abnormal immune regulation and increase susceptibility [5]. Immunologic abnormalities are the core mechanism, Th1/Th17 cell overactivation and the release of proinflammatory factors trigger chronic inflammation [6], and a defective Treg cell function further exacerbates the response [7]. When the intestinal microbiota is dysbiotic, the intestinal immune barrier function is disrupted and an abnormal immune response is activated, causing intestinal inflammation while further exacerbating the disruption of intestinal bacteria, creating a vicious cycle [8]. Furthermore, the geographic variation is related to changes in dietary structure, as well as with advances in medical technology and epidemiologic surveillance methods [9,10,11,12].

In the past, Crohn’s disease was often controlled with 5-aminosalicylates, repeated corticosteroid regimens, and immunomodulators. The widespread use of biologics has brought a new turn in the treatment of CD. Despite their efficacy, the high cost, immunogenetic nature, and parenteral administration requirements of biologics have limited their use [13]. With new therapies and changes in treatment strategies, new convenient oral agents such as JAK inhibitors have begun to emerge. With their rapid onset and their targeted mechanism, JAK inhibitors offer a promising alternative, particularly in resource-limited settings or for patients refractory to biologics [14].

This review aims to systematically elucidate the therapeutic potential of JAK inhibitors in CD. By analyzing the pathogenesis and the current status of the use of JAK inhibitors, it explores the drug applicability and safety risks, while revealing the limitations of the existing studies and providing a reference for the further application of JAK inhibitors in CD.

## 2. Pathophysiology

### 2.1. Genetic Factors

The pathogenesis of CD involves multifactorial interactions. There are two core susceptibility genes: nucleotide-binding oligomerization domain 2 (NOD2) and autophagy-associated 16-like structural domain 1 (ATG16L1) [15]. Intestinal immune cells (macrophages and dendritic cells (DCs)) express functional toll-like receptors (TLRs) and NOD-like receptors (NLRs) which bind to bacterial cell wall components and modulate the inflammatory response through two pathways. In intestinal epithelial cells, NOD2 is also expressed in Paneth cells [14]. Under normal physiological conditions, metabolites of intestinal microorganisms such as short-chain fatty acids (SCFAs), trimethylamine-N-oxide (TMAO), and branched-chain amino acids maintain intestinal homeostasis by regulating the differentiation of lymphocytes and cytokine production [16]. NOD2 recognizes cytosolic muramyl dipeptide (MDP), a small molecule derived from the peptidoglycan layer of the bacterial cell wall, which elicits an inflammatory response to maintain intestinal homeostasis [17]. When the NOD2 gene is mutated, proinflammatory factors (e.g., TNF-α, IL-6, IL-12, IL-23, etc.) are overproduced, intestinal homeostasis is lost, and the risk of CD increases exponentially [18].

Three mechanisms have been proposed to explain how mutations in the NOD2 gene lead to CD. The first is an impaired function of the Paneth cell, leading to a reduction in the production of α-defensins, which in turn leads to defective host defense against bacteria; however, there are studies that disagree on this mechanism. The second is an impaired interaction of NOD2 with ATG16L1, leading to a defective autophagic response and causing excessive inflammation. The third is an imbalance in the TLR-NOD2 co-regulatory network [17].

Loss-of-function mutations in the NOD2 gene (including Arg702Trp, Gly908Arg, and Leu1007fsinsC) are significantly and positively associated with the risk of developing CD. The NOD2 classical signaling pathway relies on the activation of serine/threonine protein kinase 2 (RIPK2) [19,20], which in turn induces the activation of NF-κB and mitogen-activated protein kinase (MAPK) and promotes the release of proinflammatory cytokines and chemokines. The activation state of RIPK2 in this process is finely regulated by site-specific ubiquitination modifications at the K63 site [18,21]. In addition, NOD2 is characterized by a multimodal function in the intrinsic immune response. In addition to classical inflammatory pathways, it can induce IFN-I production in a RIPK2-independent manner by recognizing viral single-stranded RNA and forming a complex interaction with mitochondrial antiviral-signaling protein (MAVS) [22]. Cooney et al. demonstrated that an intact NOD2-RIPK2 pathway mediates autophagy and antigen presentation in human dendritic cells in an ATG16L1-dependent manner [23]. However, mouse fibroblasts can perform these processes without relying on the RIPK2 pathway [24], which might be explained by differences in cell types.

Previous ex vivo and in vivo binding studies using rodent, human, and cellular models have shown that MDP is a degradation product of peptidoglycan (PGN) in the bacterial cell wall which activates TLR2 independently of NOD2 [25]. Watanabe et al. established a model of experimental colitis induced by the overt transfer of ovalbumin (OVA)-specific CD4+ T cells and colonization of OVA-expressing Escherichia coli, and observed that NOD2-deficient mice developed severe colitis characterized by an enhanced Th1 response [26]. Subsequently, Okai et al. induced a mouse PGN model using a NOD2 and TLR2 co-stimulation system and found that, comparing with stimulation of the TLR2 pathway alone, simultaneous stimulation of the TLR2 and NOD2 pathways enhanced the autophagic response induced by ATG16L1, resulting in decreased production of Th1 cytokines in mouse macrophages and DCs [27]. Sho Masaki et al. used in vitro cellular assays and a mouse model overexpressing interferon regulatory factor 4 (IRF4) to find that NOD2 can also be activated to induce increased expression of IRF4, which synergistically inhibits ubiquitination of the K63 linkage of RIPK2 in concert with ATG16L1, thereby suppressing the proinflammatory cytokine response caused by the TLR-RIPK pathway [28]. A mutation in NOD2 results in the absence of IRF4 and ATG16L1 activation, which enhances TLR-mediated NF-κB activation and proinflammatory cytokine responses, ultimately contributing to the development of CD.

### 2.2. Immune System Response Disorders

Intestinal immunity in a healthy intestine consists of two types, intrinsic and adaptive immunity. Intrinsic immunity refers to the barrier function of the intestinal mucosa, antimicrobial proteins, gastric acid, intrinsic immune cells (neutrophils, macrophages, DCs, and NK cells, among others), as well as innate cytokines and molecules (IL-1 and defensins) [28]. Adaptive immunity is usually initiated when intrinsic immunity fails to clear pathogens, and is achieved primarily through T and B cells [29]. When intestinal immunity is dysfunctional, this will lead to the development of CD [30].

The intestinal epithelial barrier consists mainly of a monolayer of intestinal epithelial cells (IEC), which can be divided into several units with different functions. For example, the goblet cells secrete a protective mucus layer, whereas the Paneth cells, which are located in the crypts of the small intestine, secrete antimicrobial peptides such as defensins, cathelicidins, and calreticulin. The intact intestinal epithelium maintains intestinal homeostasis by restricting antigenic entry into the mucosa, providing TLRs and NLRs, participating in the construction of an intestinal tolerogenic environment, and controlling the immune system in the intestinal-associated lymphoid tissues [31]. Disruption of the intestinal epithelial barrier, often caused by genetic mutations (e.g., CDH1, HNF4A, LAMB1) or hyperpermeability induced by elevated proinflammatory cytokines (such as TNF-α and IFN-γ), is a key mechanism in the development of intestinal inflammation [32]. In addition, an increased rate of apoptosis and decreased regeneration of intestinal epithelial cells is often observed in patients with CD, further disrupting the intestinal barrier and leading to prolonged inflammation [32].

In adaptive immunity, dendritic cells are antigen-presenting cells that drive the release of proinflammatory factors by upregulating TLRs expression in response to inflammatory stimuli [33]. In CD, dendritic cells are abnormally activated to release proinflammatory factors, such as IL-6 and IL-12, to drive the immune response of Th1 cells, whose function is often influenced by factors such as the expression of NOD2, IRF4, and TLR [34]. Meanwhile, macrophages in the intestinal mucosa are responsible for the removal of apoptotic or senescent cells as well as the reconstitution of cellular tissues. Activated macrophages typically express high levels of costimulatory factors and release large amounts of IL-12 and IL-23, forming a positive feedback pathway for proinflammatory signaling [35]. Innate lymphocytes (ILCs) are a heterogeneous population of cells that are closely associated with the maintenance of intestinal barrier integrity. Upon stimulation, they produce cytokines such as TNFα, IL-17, IL-22, and IFN-γ to exacerbate mucosal injury [36].

CD4^+^ Th cells play an important role in adaptive immunity in the face of microbial infections and can differentiate into Th1, Th2, Treg, Th17, Tfh, and Th9 cells upon stimulation with different cytokines [7]. CD is often thought to be closely related to overactivity of Th1 and Th17 cells. In the intestinal mucosa of patients with CD, the Th1 cytokine IFN-γ is significantly increased [25]. Animal models have also demonstrated that removal of IFN-γ is effective in preventing colitis. Flow cytometry analysis of mucosal cells revealed an increased number of IL-17-producing T cells in patients with CD compared to healthy controls [37]. In addition, impaired suppression of intestinal Treg cells has also been associated with the pathogenesis of CD [38]. Studies using an animal model of colitis found that Treg cells can reduce intestinal inflammation by downregulating Th1 and Th17 responses. Animal experiments further revealed that the mechanism by which Treg inhibit intestinal inflammation through the secretion of IL-10, TGF-β, and IL-35 has a dose-dependent character [38,39,40]. Additionally, the excessive production of nonspecific inflammatory mediators like TNF, TL1A, and IL-6 increases the risk of CD [41,42]. This association is supported by the clinical use of anti-TNF and anti-IL-6R monoclonal antibodies.

### 2.3. Dysbiosis of Intestinal Microbiota

After birth, the human gastrointestinal tract develops a complex microbial community characterized by significant biodiversity, primarily consisting of the *phyla Firmicute*, *Bacteroidete*, and a major producer of SCFAs (e.g., butyric acid), which are essential for maintaining the intestinal barrier and anti-inflammatory [16]. In the immune response, the immune system specifically recognizes pathogen-associated molecular patterns (PAMPs) carried by microorganisms through pattern recognition receptors (PRRs, including TLRs, NLRs, C-type lectin receptors and RIG-I-like receptors, etc.). Initiation of the immune response cascade elicits NF-κB-signaling pathways and inflammatory vesicle activation mediating the synthesis and release of proinflammatory cytokines and chemokines [43].

The gut microbiota plays a key regulatory role in maintaining intestinal homeostasis. Dysbiosis of the microbiota, caused by factors such as drugs, diet, smoking, and acute infections, results in abnormal expression of tight junction proteins in intestinal epithelial cells, increasing permeability [8]. Large cohort studies have shown that in active CD, intestinal dysbiosis is characterized by reduced diversity, metabolic dysfunction and imbalance in flora dynamics. In particular, there is a decrease in the following anti-inflammatory bacteria: SCFA-producing *F. prausnitzii*, *Roseburia* and *Ruminococcus*, proinflammatory bacteria such as adherent invasive *Escherichia coli* (AIEC), *Ruminococcus gnavus*, *Enterobacteriaceae* and *Fusobacterium* and other over-colonizing bacteria [44]. Serologic studies further revealed elevated levels of anti-*Saccharomyces cerevisiae* mannan antibody (ASCA) in CD, and elevated antibody titers often preceded the onset of clinical symptoms, suggesting a potential association of fungi (e.g., Saccharomyces *cerevisiae*) with IBD [45]. Dysbiosis promotes translocation of microorganisms and their metabolites to the lamina propria, while aberrant immune activation promotes the release of large quantities of proinflammatory cytokines [46], culminating in the formation of a vicious cycle of “inflammation-dysbiosis” [43]. To address this, interventions such as probiotics and fecal transplants are currently available to restore flora balance [47]. Dietary therapies (e.g., low-sugar, low-fat, and high-fiber) have also been shown to improve immune abnormalities and intestinal flora disorders in CD to some extent [48,49,50].

## 3. The Use of JAK Inhibitors in Crohn’s Disease

Pharmacologic strategies for Crohn’s disease have undergone a paradigm shift from traditional immunomodulators to biologics to novel oral small molecule drugs (SMDs) including JAK inhibitor and sphingosine-1-phosphate (S1P) modulators [51]. Acetylsalicylic acid analogs, corticosteroids, and immunosuppressive agents such as azathioprine and methotrexate are often used in conventional therapy to induce or maintain clinical remission in CD. In recent years, the widespread use of biological agents such as anti-TNF drugs and anti-IL drugs has significantly improved clinical outcomes [13]. However, such biologics have obvious limitations: they need to be administered intravenously or subcutaneously, long-term use of biologics may increase the risk of opportunistic infections and malignant tumors [52,53]. The therapeutic effects for patients with long-standing moderate-to-severe CD often do not meet expectations due to initial non-responsiveness and immunogenicity. A cohort study by Chanchlani et al. showed that only about 33% of patients receiving anti-TNF drugs (e.g., adalimumab, infliximab) for active tubulointerstitial CD maintained remission after three years of treatment [54]. Kennedy et al. observation of 1610 patients treated with anti-TNF therapy further confirmed that 24% of patients experienced primary nonresponse and 63% did not achieve clinical remission [55].

In response to the limitations of anti-TNF therapy, novel gut-selective agents such as vedolizumab, a gut-selective anti-α4β7 integrin monoclonal antibody, have demonstrated a superior safety profile, but their mechanism of action limits their use in extra-intestinal lesions and the evidence for long-term efficacy is insufficient. Other biologics such as ustekinumab and risankizumab have achieved endoscopic improvement and remission of moderate-to-severe CD in randomized controlled trials compared with placebo, but again, there is a lack of long-term safety data to support this [56,57,58]. In response to the above challenges, the research hotspots have gradually shifted to small molecule drugs with the advantage of oral administration, among which JAK inhibitors have attracted much attention due to their non-immunogenicity, short half-life, and rapid onset of action [59].

**Figure 1:**
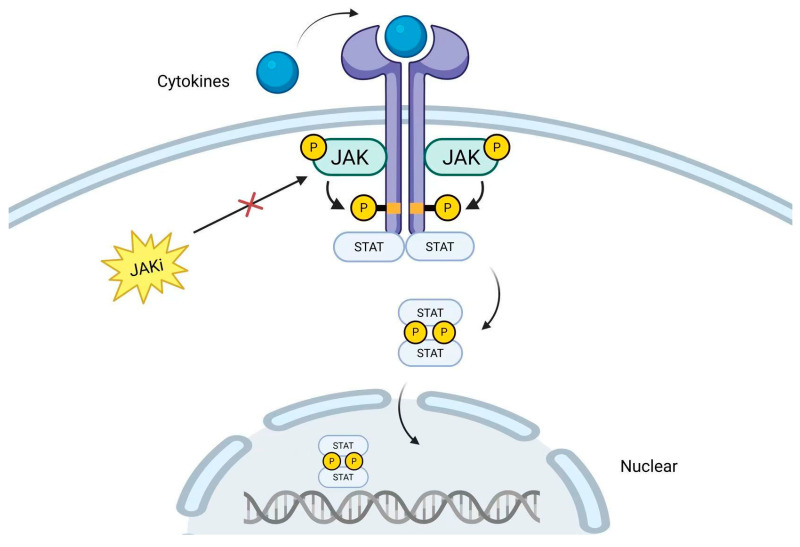
Mechanism of JAK inhibitors.

The JAK/STAT signaling pathway plays a key role in the pathogenesis of CD as a core transduction pathway for cytokine-mediated immune responses [59]. When a ligand such as cytokines binds to a membrane receptor, the receptor dimerizes and activates the associated JAK kinase, inducing autophosphorylation of JAK and phosphorylation of tyrosine residues on the receptor. The phosphorylated receptor recruits cytoplasmic STAT proteins, which are phosphorylated by JAK, form dimers, and translocate into the nucleus, triggering downstream inflammatory responses [60] (Figure 1).

The JAK family comprises JAK1, JAK2, JAK3, and TYK2, with heterodimers such as JAK1/JAK2 and JAK1/JAK3 mediating signals for IL-6, IL-12/IL-23, and γ-chain cytokines (e.g., IL-2, IL-7) [61]. JAK1 primarily inhibits the signaling of proinflammatory cytokines, thereby reducing Th1/Th17-driven inflammatory responses. Highly selective JAK1 inhibitors significantly interfere with the IFN-γ pathway, leading to diminished antiviral capacity and increased risk of opportunistic infections such as herpes zoster [60]. JAK2 is associated with fibroblast growth factor receptor (FGFR), vascular endothelial growth factor receptor (VEGFR), and platelet-derived growth factor receptor (PDGFR) pathways, regulating immune responses, mediating hematopoietic stem cell signal transduction, and influencing erythrocyte and thrombus production [62,63]. JAK3 governs signal transduction of the γ-chain cytokine family members and is critical for early hematopoietic stem cell growth/differentiation and immune regulation [64]. TYK2 participates in IL-12, IL-23, and IFN-I signaling, and is indispensable for NK cell activity, B-cell maturation, and Th1/Th17 cell differentiation [65].

The STAT family comprises seven structurally and functionally related proteins, each corresponding to specific cytokines to regulate distinct genes. STAT1 serves as an essential transcription factor for Th1/Th17 cell differentiation and participates in multiple interferon signaling pathways [66]; STAT2 primarily mediates IFN-I-associated signaling, though its mechanism in IBD remains unclear [67]. STAT3 is activated by diverse cytokines and plays significant roles in controlling inflammation, viral infections, and antitumor immunity [68]. STAT4 mediates IFN-I and IL-12 signaling, and is implicated as a pathogenic factor in Th1/Th17-mediated autoinflammatory diseases [69]. STAT5 (including STAT5a/STAT5b) predominantly regulates CD4+ T-cell amino acid metabolism and promotes regulatory T-cell development [70]. STAT6, primarily activated by IL-4 and IL-13, participates in Th2-mediated immunity and is crucial for intestinal parasite clearance and allergic disorders [71].

In CD, Th1/Th17 cell overactivation drives excessive release of IL-12, IL-23, and IFN-γ, perpetuating JAK-STAT signaling and intestinal barrier disruption [72]. Selective JAK inhibitors competitively bind to the ATP-binding domain of JAKs, blocking STAT phosphorylation and nuclear translocation, which downregulates transcription of inflammatory mediators like TNF-α and matrix metalloproteinases (MMPs) [73]. Currently, several JAK inhibitors have been developed, and representative drugs, such as tofacitinib, upadacitinib, and filgotinib, have been entered into clinical trials.

### 3.1. Tofacitinib

Tofacitinib is a JAK1 and JAK3 inhibitor that is less effective against JAK2 [74]. It was the first JAK inhibitor approved for moderately to severely active ulcerative colitis (UC) in patients who had previously responded poorly to conventional therapy and has demonstrated excellent efficacy and safety in both UC and rheumatoid arthritis (RA) [75,76,77]. Tofacitinib is metabolized by the cellular chromogranin CYP3A4 metabolism, has a rapid onset of action and short half-life when administered orally, and requires twice-daily dosing [78].

In a randomized, double-blind, placebo-controlled phase 2 trial, there was no significant difference in the percentage of patients with moderately severe active CD who achieved a clinical response (≥70-point reduction in CDAI from baseline) or clinical remission after 4 weeks of twice-daily tofacitinib (1, 5, or 15 mg) or placebo [79]. In another randomized double-blind trial, at week 8 of induced remission, the proportion of patients in clinical remission was 43.5% and 43.0% in patients receiving tofacitinib twice daily (5 mg and 10 mg) versus 36.7% in the placebo group (*p* = 0.325 and *p* = 0.392). The proportion of patients in clinical response (CDAI-100) or clinical remission was higher in patients receiving tofacitinib 10 mg bid at week 26 of induction (55.8% vs. 38.1%, *p* = 0.130), but none of the differences were statistically significant [76] (Table 1).

Previous studies have demonstrated the significant efficacy of tofacitinib in moderate-to-severe UC, attributed to Th2-dominated mucosal inflammation driven by key cytokines such as IL-13, IL-5, and IL-9, which rely on JAK1/JAK3 heterodimers for signal transduction [80]. In contrast, CD is associated with Th1/Th17-driven immune responses mediated by cytokines including IL-12, IL-23, and IFN-γ, whose signaling depends on JAK2/TYK2 heterodimers. However, tofacitinib exhibits minimal inhibitory effects on JAK2 and TYK2, explaining its limited therapeutic impact in CD [72].

### 3.2. Filgotinib

Filgotinib is a selective JAK1 inhibitor, and preferential inhibition of JAK1 modulates a specific subpopulation of proinflammatory cytokines in the JAK-STAT pathway, which differentiates it from the types of cytokines acted upon by JAK2 or JAK3 inhibition [81]. It demonstrates an approximately 30-fold higher selectivity for JAK1 over JAK2 and about 50-fold greater selectivity for JAK1 compared to JAK2 in human whole blood, and has shown clinical efficacy in moderate-to-severe active UC [82]. Filgotinib is widely and rapidly absorbed after oral administration, and maximal pharmacodynamic effect achieved when administered at 200 mg per day [83].

In a 2017 randomized, double-blind, placebo-controlled phase 2 trial, 174 patients with active CD were randomized to receive either once-daily filgotinib 200 mg or placebo for 10 weeks. The primary endpoint of the trial was clinical remission (defined as a CDAI of less than 150 at week 10). At week 10, the percentage of the treatment group achieving clinical remission was 47% (vs. 23% in the placebo group, *p* < 0.01). However, in terms of endoscopic remission, the difference between the treatment and placebo groups was not statistically significant. This may be related to the fact that CD usually presents as transmural ulcers, which require a longer duration of treatment before meaningful endoscopic improvement may be observed. Thus, even though filgotinib has shown some efficacy in clinical remission of CD, further studies are needed [84] (Table 1).

A recently published phase 3 DIVERSITY clinical trial, which was multicenter, double-blind, randomized, and placebo-controlled, evaluated the efficacy and safety of filgotinib for treating CD. The trial was divided into two induction studies, with patients who had not been treated with biologics and those who had been treated with biologics included in induction trial A. Patients who had failed biologic therapy included in induction trial B. Subjects were randomized to receive once-daily filgotinib 200 mg, 100 mg or placebo for 10 weeks, followed by a maintenance phase for those who achieved PRO2 clinical remission (abdominal pain score ≤ 1 and bowel movement character score ≤ 3) or endoscopic response (simplified endoscopic score [SES-CD] ≥ 50% reduction from baseline) at week 11, with continued observation until week 58. The primary endpoints were PRO2 clinical remission and endoscopic response at weeks 10 and 58. Results showed that during induction, the 200 mg group demonstrated significant superiority in Study B (PRO2 remission: 29.7% vs. 17.9%, *p* = 0.0039) but not in Study A (32.9% vs. 25.7%, *p* = 0.0963). Neither of them showed significant endoscopic improvement during induction. Whether this indicates that refractory CD patients who have failed biologic therapy are more sensitive to filgotinib still warrants further detailed investigation. In the maintenance phase, the 200 mg group outperformed placebo in PRO2 remission (43.8% vs. 26.4%, *p* = 0.0382) and endoscopic response (30.4% vs. 9.4%, *p* = 0.0038). The 100 mg dose failed to meet primary endpoints in all studies [85].

### 3.3. Upadacitinib

Upadacitinib is a selective JAK1 inhibitor, demonstrating an approximately 40-fold higher selectivity for JAK1 over JAK2 and over 100-fold greater selectivity compared to JAK3. It has been approved for treating CD, UC, rheumatoid joints, and atopic dermatitis [86]. Upadacitinib is metabolized predominantly by CYP3A4 and, to a lesser extent, by CYP2D6, and hepatic insufficiency and renal insufficiency have no clinically relevant effect on the concentration on it, which is significantly affected in the organism only by interactions with the other substrates of CYP3A4 [87].

A double-blind dose-ranging phase 2 trial evaluated the efficacy and safety of upadacitinib in patients with moderate-to-severe CD who had failed prior biologic therapy. This trial was divided into an induction phase lasting 16 weeks and a maintenance remission phase lasting 36 weeks. A total of 220 subjects were enrolled and randomly assigned to six intervention groups (3 mg, 6 mg, 12 mg, 24 mg bid, and 24 mg once daily) or a placebo group. The co-primary endpoints were clinical remission at week 16 (defined as mean daily stool frequency ≤ 1.5 and abdominal pain score ≤ 1.0) and endoscopic remission at week 12/16 (defined as simple endoscopic score of CD (SES-CD) ≤ 4 or a reduction of ≥2 points from baseline without a single score > 1). The results of the study showed that at the induction endpoint, clinical remission rates ranged from 11 to 27% (vs. 11% in the placebo group) across the dose groups, with a statistically significant difference in the results in the 6 mg bid group only (*p <* 0.1), and no clear dose–response relationship was observed. Endoscopic remission, on the other hand, showed a more pronounced dose–response relationship, with a remission rate of 22% (*p <* 0.01 vs. 0% in the placebo group) in the highest-dose group (24 mg bid), compared with 8–14% in the other active drug groups. The proportion of patients receiving corticosteroids at baseline who achieved hormone withdrawal with a Crohn’s Disease Activity Index (CDAI) < 150 at week 16 was significantly higher in the 12 mg versus 24 mg bid group than in the placebo (both *p <* 0.05) [88] (Table 1).

Based on the results of the phase 2 study, three subsequent phase 3 studies (U-EXCEL/U-EXCEED induction trial and U-ENDURE maintenance trial) were conducted with optimized dosing regimens. During the 12-week induction period, patients with moderate-to-severe CD were randomized to receive Upadacitinib 45 mg qd or placebo. In the subsequent maintenance trial (U-ENDURE), patients who had a clinical response in the induction trial were randomized to upadacitinib 15 mg, 30 mg once-daily, and placebo for 52 weeks. The primary endpoints were clinical remission (defined as CDAI < 150) and endoscopic response (>50% reduction from baseline in SES-CD or 2-point reduction in patients with baseline SES-CD ≥ 4). At week 12, results indicated that patients receiving 45 mg of upadacitinib had significantly higher rates of clinical remission compared to the placebo group: 49.5% vs. 29.1% in U-EXCEL, and 38.9% vs. 21.1% in U-EXCEED. Additionally, there was a significant difference in endoscopic response rates: 45.5% vs. 13.1% in U-EXCEL, and 34.6% vs. 3.5% in U-EXCEED (all *p* < 0.001). During the maintenance period, patients who responded to induction treatment continued receiving either 15 mg or 30 mg of upadacitinib daily, demonstrating sustained benefits that were dose-dependent. The clinical remission rates and endoscopic response rates were significantly higher in the 30 mg group (47.6% and 40.1%, respectively) compared to the 15 mg group (37.3% and 27.6%) and the placebo group (15.1% and 7.3%) (all *p* < 0.001 for group comparisons) [86]. (Table 1).

Given the clinical efficacy of upadacitinib in random clinical trials, real-world evidence has become an important extension of current research. A number of real-world studies have been initiated globally with the aim of evaluating the long-term efficacy, safety and cost-effectiveness profile of the drug in real clinical situations [89,90,91,92,93,94,95,96] (Table 2).

Although these data indicate that JAK inhibitors are more effective than placebo, its superiority or non-inferiority compared to approved biologics (such as infliximab and risankizumab) has not been confirmed by direct comparative trials. Future studies should conduct cross-mechanism direct comparative trials to clarify the advantageous positioning of JAK inhibitors in biologic-experienced or biologic-naïve populations.

### 3.4. Other JAK Inhibitors

A number of JAK inhibitors are still being investigated. Izencitinib (TD-1473) is a pan-JAK inhibitor whose phase 1 clinical trial against UC was completed; however, its phase 2 trial against CD (NCT03635112) was terminated. The exact reason for the termination was not clearly stated in the available information, but it is speculated that it may be related to safety issues that arose during the course of the trial, efficacy that did not meet expectations, etc. Ritlecitinib (PF-06651600), a JAK3/TEC family kinase inhibitor [97], and Brepocitinib (PF-06700841), a TYK2/JAK1 inhibitor [98] have shown efficacy and safety in pemphigus vulgaris and rheumatoid arthritis [99,100]. A phase 2 trial in CD (NCT03395184) has now been completed. Ivarmacitinib, a selective JAK1 inhibitor previously known as SHR0302, has been shown to be efficacious and well tolerated in ulcerative colitis in a randomized, double-blind, placebo-controlled, phase 2 trial [101]. Currently, a phase 2 study (NCT03677648) is ongoing to further explore its potential in treating Crohn’s disease. Deucravacitinib, also known as BMS-986165, is a highly selective oral TYK2 inhibitor that has shown therapeutic efficacy in inflammatory bowel disease in the animal testing phase [102]. In 2022 it was approved for the treatment of moderate to severe plaque psoriasis in adults [103], and it has now completed phase 2 clinical trials in CD(NCT04877990, NCT03599622).

## 4. Safety of JAK Inhibitors

Although JAK inhibitors have shown potential in the treatment of multiple autoimmune diseases by inhibiting immune responses through targeted modulation of the JAK-STAT signaling pathway and have filled a gap in the existing treatment options for IBD, their long-term safety needs to be further validated. Between 2019 and 2021, FDA issued the highest level of black-box warnings for such drugs, which clearly states their potential for increased risk of death, major adverse cardiovascular events (MACE), venous thromboembolism (VTE), malignancy, and serious infections, among other safety concerns. This warning emphasizes the need for careful evaluation of the risk–benefit ratio of JAK inhibitors in clinical use.

### 4.1. Infectious

The broad-spectrum immunosuppressive effects of JAK inhibitors may increase the risk of opportunistic infections. Data from the phase 2 clinical trial of Tofacitinib showed that the most common infectious adverse events (AEs) in patients with UC were influenza (*n* = 6) and nasopharyngitis (*n* = 6), with two patients experiencing serious infectious AEs [104]. Post-marketing surveillance further found that the estimated rate of serious infections reported in patients with UC (3.28/100 PYs) was significantly higher than in patients with RA (2.57/100 PYs). The most common infections were nasopharyngitis (134 cases) and herpes zoster (HZ) (127 cases), while serious infectious events were dominated by Clostridium difficile infection (51 cases), pneumonia (36 cases), COVID-19 (12 cases), cytomegalovirus infection (8 cases), and HZ (8 cases) [105]. In addition, other JAK inhibitors have shown differences in infection risk: the incidence of HZ was lower with filgotinib, but showed a dose-dependent pattern (5 cases in the 200 mg group vs. 1 case in the 100 mg group) [99,106]. In a phase 3 trial of upadacitinib, 3 cases of severe HZ events were reported in the 45 mg once-daily dosage group [107]. Combining data from multiple phase 2/3 trials, JAK inhibitors as a whole showed similar trends in HZ risk [108].

### 4.2. Major Adverse Cardiovascular Events

The ORAL Surveillance study targeted patients with ≥1 cardiovascular risk factor (e.g., smoking, hypertension, hyperlipidemia, diabetes mellitus, or a history of cardiovascular disease) who were randomly assigned to tofacitinib (5 mg twice-daily, 10 mg twice-daily) or a TNF inhibitor group. The results showed that neither dose of tofacitinib met the noninferiority criterion for MACE: the incidence of MACE was 0.91/100 PYs in the 5 mg group and 0.73/100 PYs in the TNF inhibitor group (HR 1.24; 95%CI 0.81–1.91) [109]. However, in patients with UC, the clinical trials and long-term follow-up in real-world cohorts, no increased risk of MACE, VTE, or malignancy was observed in the tofacitinib group [110]. Further comparative studies showed no significant difference in the incidence of MACE between the JAK inhibitor (including baricitinib and tofacitinib) group and the adalimumab group (54 vs. 35 patients, HR 1.0; 95%CI 0.7–1.5, *p* = 0.99) [111]. A phase 3 trial for upadacitinib found that the incidence of MACE was 0.3, 0.3, and 0.2/100 PYs in the 15 mg once-daily group, the adalimumab group, and the methotrexate (MTX) monotherapy group, respectively, and that the risk increased with age and the accumulation of cardiovascular risk factors (e.g., smoking, hypertension) [112]. In the case of upadacitinib for the treatment of UC in the phase 2b/3 trials (U-ACHIEVE induction and U-ACCOMPLISH), MACE occurred in only 1 patient in the 30 mg group and 1 patient in the placebo group, both of whom had known cardiovascular risk factors [113]. Thus, there is some population heterogeneity with respect to cardiovascular risk with JAK inhibitors that needs to be further evaluated.

### 4.3. Thrombosis

Studies have shown that the incidence of venous thromboembolism (VTE) risk was comparable to that of TNF inhibitors in the low-dose group (5 mg twice-daily) of tofacitinib (0.3 vs. 0.2/100 PYs), but the risk was significantly higher in the high-dose group (10 mg twice-daily) (0.8/100 PYs) [114]. An analysis of a study that included 12,410 patients with RA, psoriatic arthritis, psoriasis, and IBD (data sources included the FDA Adverse Event Reporting System and the IBD Market Scan database) treated with tofacitinib showed that the incidence of deep vein thrombosis (DVT), pulmonary embolism (PE), and arterial thrombotic events was significantly higher in patients with underlying cardiovascular risk factors than in those without, with the latter having very low rates [115]. Notably, upadacitinib demonstrated a similar trend toward thromboembolic risk as tofacitinib in clinical trials, but further long-term studies are needed [106]. Whereas phase 3 studies and long-term follow-up data for filgotinib showed no venous thrombosis or pulmonary embolism events were observed in its treatment group [85]. Additional date of long-term use of filgotinib for the treatment of immune disorders also suggest no significant risk of thrombotic events with filgotinib [116]. Overall, caution remains warranted when using JKA inhibitors in patients with risk factors for thrombosis.

### 4.4. Malignant Tumor

Long-term use of JAK inhibitors may pose a risk of malignancy. ORAL Surveillance data showed that the risk of malignancy was higher in the tofacitinib 5 mg vs. 10 mg twice-daily group than in the TNF inhibitor group (HR = 1.13, 95% CI 0.87–1.48 vs. HR = 1.13, 95% CI 0.86–1.48; HR = 0.77, 95% CI 0.55 in the TNF inhibitor control group) [109]. However, the available data did not show a significant difference in the mortality rate of malignancies between marketed JAK inhibitors and biologics [117]. A pooled analysis of tofacitinib found a malignancy incidence rate of <1 case/100 PYs in 1157 patients with UC on long-term therapy (median duration 1.7 years) [76]. Other JAK inhibitors have clinical data that are similarly suggestive of low risk: no malignancy events were reported in both the UC induction and maintenance trials of filgotinib [84,85]. In randomized controlled trials of upadacitinib for UC, 1 malignancy event was reported during the induction phase and 2 malignancy events were reported during the maintenance phase [107]. However, regardless of the type of JAK inhibitor, the development of malignancy is a rare event that still needs to be studied in the long term.

Although head-to-head randomized trials are lacking, current evidence suggests that JAK inhibitors exhibit comparable infection risks and cardiovascular safety to TNF inhibitors, but may carry higher risks of malignancy and venous thrombosis (e.g., with tofacitinib), particularly in long-term users or high-risk populations [118,119,120,121]. Therefore, the clinical application of JAK inhibitors should be guided by dynamic risk stratification assessment. Priority should be given to patients who have failed prior biologic therapy and exclude those with high-risk factors for cardiovascular or venous thromboembolic events. The use of next-generation highly selective JAK inhibitors is recommended, followed by the implementation of personalized treatment regimens and regular monitoring for comprehensive management. Future research should focus on extending follow-up periods and expanding real-world data collection to refine long-term risk stratification strategies.

## 5. Conclusions

Crohn’s disease is a complex disease characterized by chronic transmural inflammation of the gastrointestinal tract, the pathogenesis of which involves multiple interactions between genetic susceptibility, immune dysregulation, disturbances in intestinal flora and environmental factors.

Unlike UC, which mainly involves the mucosal layer, the intestinal inflammation of CD that involves the entire thickness of the intestinal wall determines that its treatment goals should place more emphasis on endoscopic remission rather than merely the improvement of clinical symptoms. Studies have shown that endoscopic remission is significantly associated with the long-term prognosis of CD patients, while the remission of clinical symptoms may only reflect the temporary control of superficial inflammation [122].

Currently, among the JAK inhibitors, tofacitinib, filgotinib, and upadacitinib all show potential in the treatment of inflammatory bowel disease. In the induction treatment phase of filgotinib, although it can significantly improve the clinical symptoms of CD, the endoscopic remission rate did not reach statistical significance. However, in the maintenance study extended to 52 weeks, a significant increase in endoscopic remission was observed (30.4% vs. 9.4%), suggesting that this drug requires long-term treatment to achieve deep control of transmural inflammation. In contrast, upadacitinib performed particularly well in phase 3 trials, with a significantly better clinical remission rate than placebo in the induction phase (49.5% vs. 29.1%), and a continuous improvement in the rate of endoscopy response during the maintenance treatment period (40.1% vs. 7.3%).

However, these studies generally had limitations: small sample sizes, short follow-up times and lack of head-to-head comparative data with existing biologics. Furthermore, the magnitude of improvement in the primary endpoint has not been sufficient to demonstrate clinical benefit. In addition, the safety profile of JAK inhibitors needs to be carefully evaluated, with risks of class effects including opportunistic infections, thrombotic events, and potential malignancy, especially at high doses or in long courses. Several clinical trials of novel JAK inhibitors are currently underway with the aim of further clarifying their risk-benefit ratio [123].

In the future, the incidence and prevalence of CD is expected to increase significantly with accelerated urbanization, innovations in medical technology and standardization of diagnostic criteria. The clinical application of existing therapeutic means such as biologics and SMDs still needs to improve the safety assessment system through the accumulation of long-term real-world data and the construction of post-marketing pharmacovigilance system. It is noteworthy that the development of new targeted drugs (such as TYK2 inhibitor Deucravacitinib, SMDs, etc.) and their combined therapeutic regimens have opened up new avenues for precision treatment strategies for CD. Overall, JAK inhibitors have provided an important breakthrough, but their clinical translation needs to be carefully weighed between efficacy and safety for long-term patient benefit.

## Figures and Tables

**Table 1:** Randomized controlled trials of JAK inhibitors in Crohn’s disease.

Drug	Trial	Dose	Patients (*n*=)	Clinical Release	Mucosal Healing	Adverse Events	Serious Adverse Events
Tofacitinib	Phase 2, RCT	Induction therapy for 8 weeks: Tofacitinib 5 mg BID, 10 mg BID vs. Placebo	280	Tofacitinib (5 mg, 10 mg vs. Placebo) 43.5%, 43.0% vs. 36.7% (*p* = 0.325 vs. *p* = 0.392)	-	Tofacitinib (5 mg) 58.1% (10 mg) 60.5% Placebo 60.4%	Tofacitinib (5 mg) 3.5% (10 mg) 11.6% Placebo 3.3%
Filgotinib	Phase 2, RCT	Induction therapy for 10 weeks: Filgotinib 200 mg QD vs. Placebo	174	Filgotinib vs. Placebo 47% vs. 23% (*p* = 0.0077)	Filgotinib vs. Placebo 25% vs. 14% (*p* = 0.31)	Filgotinib 75% Placebo 67%	Filgotinib 14% Placebo 4%
Filgotinib	Phase 3, RCT, DIVERSITY	Induction therapy A for 10 weeks: Filgotinib 200 mg QD, 100 mg QD vs. Placebo	707	Filgotinib (100 mg, 200 mg vs. Placebo) 29.8%, 32.9% vs. 25.7% (*p* = 0.3050 vs. *p* = 0.0963)	Filgotinib (100 mg, 200 mg vs. Placebo) 18.4%, 23.9% vs. 18.1% (*p* = 0.5103 vs. *p* = 0.1365)	Filgotinib (100 mg) 56% (200 mg) 51% Placebo 58%	Filgotinib (100 mg) 7% (200 mg) 8% Placebo 6%
		Induction therapy B for 10 weeks: Filgotinib 200 mg QD, 100 mg QD vs. Placebo	665	Filgotinib (100 mg, 200 mg vs. Placebo) 18.9%, 29.7% vs. 17.9% (*p* = 0.7556 vs. *p* = 0.0039)	Filgotinib (100 mg, 200 mg vs. Placebo) 13.6%, 11.9% vs. 11.4% (*p* = 0.4264 vs. *p* = 0.9797)	Filgotinib (100 mg) 68% (200 mg) 70% Placebo 68%	Filgotinib (100 mg) 16% (200 mg) 9% Placebo 11%
		Maintenance therapy for 46 weeks: Filgotinib 100 mg to 00 mg, 100 mg to Placebo, 200 mg to 200 mg, 200 mg to Placebo, Placebo to Placebo	481	Filgotinib (200 mg to 200 mg vs. 200 mg to Placebo) 43.8% vs. 8.9% (*p* = 0.0382) *	Filgotinib (200 mg to 200 mg vs. 200 mg to Placebo) 30.4% vs. 8.9% (*p* = 0.0038) *	Filgotinib (100 mg to Placebo) 65% (100 mg to 100 mg) 72% (200 mg to Placebo) 63% (200 mg to 200 mg) 68% Placebo to Placebo 66%	Filgotinib (100 mg to Placebo 5% (100 mg to 100 mg) 13% (200 mg to Placebo) 9% (200 mg to 200 mg) 11% Placebo to Placebo 10%
Upadacitinib	Phase 2, RCT	Induction therapy for 16 weeks: Upadacitinib 3mg BID, 6 mg BID, 12 mg BID, 24 mg BID, 24 mg QD vs. Placebo	220	Upadacitinib (6 mg BID vs. Placebo) 27% vs. 11% (*p* < 0.01) *	Upadacitinib (24 mg BID vs. Placebo) 22% vs. 0 (*p* < 0.01) (24 mg QD vs. Placebo) 14% vs. 0 (*p* < 0.05) *	Upadacitinib (3mg bid) 87.2% (6 mg bid) 78.4% (12 mg bid) 80.6% (24 mg bid) 83.8% (24 mg qd) 82.9% Placebo 73%	Upadacitinib (3mg bid) 12.8% (6 mg bid) 5.4% (12 mg bid) 27.8% (24 mg bid) 8.3% (24 mg qd) 20.0% Placebo: 5.4%
Upadacitinib	Phase 3, RCT, U-EXCEL	Induction therapy for 12 weeks: Upadacitinib 45 mg QD vs. Placebo	526	Upadacitinib vs. Placebo 49.5% vs. 29.1% (*p* < 0.001)	Upadacitinib vs. Placebo 45.5% vs. 13.1 (*p* < 0.001)	Upadacitinib 62.6% Placebo 58.5%	Upadacitinib 8.9% Placebo 8.5%
	U-EXCEED	Induction therapy for 12 weeks: Upadacitinib 45 mg QD vs. Placebo	495	Upadacitinib vs. Placebo 38.9% vs. 21.1% (*p* < 0.001)	Upadacitinib vs. Placebo 34.6% vs. 3.5% (*p* < 0.001)	Upadacitinib 68.2% Placebo 65.5%	Upadacitinib 8.6% Placebo 11.7%
	U-ENDURE	Maintenance therapy for 40 weeks: Upadacitinib 15 mg QD, 30 mg QD vs. Placebo	502	Upadacitinib (15 mg QD, 30 mg QD vs. Placebo) 37.3%, 47.6% vs. 15.1% (*p* < 0.001 vs. *p* < 0.001)	Upadacitinib (15 mg QD, 30 mg QD vs. Placebo) 27.6%, 40.1% vs. 7.3% (*p* < 0.001 vs. *p* < 0.001)	Upadacitinib (15 mg) 83.3% (30 mg) 78.7% Placebo 74.6%	Upadacitinib (15 mg) 10.1% (30 mg) 13.1% Placebo 12.1%

RCT: Randomized Controlled Trial; QD: Once daily; BID: Twice daily; *: Only the parts with statistically significant *p*-values are listed in the table.

**Table 2:** Real-world research of JAK inhibitor in Crohn’s disease.

Drug	Dose	Patients (*n*=)	Clinical Release	Mucosal Healing	Adverse Events	Serious Adverse Events
Tofacitinib	Induction therapy for 8 weeks: 10 mg BID	76	46.6% (35/76)	44.8% (34/76)	17.1% (13/76)	7.9% (6/76)
Upadacitinib	Induction therapy for 12 weeks and maintenance therapy for 12 weeks: 15 mg QD, 30 mg QD, 45 mg QD	45 (efficacy cohort: 33)	CR at week 12 27.3% (9/33)	MH at week 24 28.6% (4/14)	27% (12/45)	4.4% (2/45)
Upadacitinib	Induction therapy for 20 weeks: 15 mg QD	12	25% (3/12)	41.67% (5/12)	58.3% (7/12)	0 (0/12)
Upadacitinib	Induction therapy for 8 weeks: 45 mg QD	105 (efficacy cohort: 88)	70.6% (12/17)	-	32.4% (34/105)	0.9% (1/105)
Upadacitinib	Induction therapy for 12 weeks: 45 mg	22	90% (18/20)	30% (6/20)	15.7% (3/22)	0 (0/22)
Upadacitinib	Induction therapy for 12 weeks: 45 mg QD Maintenance therapy for 40 weeks: 15 mg QD, 30 mg QD	246	CR at week 52 79.2% (19/24)	MH at week 52 54.5% (6/11)	46.8% (101/215)	0 (0/215)
Upadacitinib	Induction therapy for 12 weeks: 45 mg QD Maintenance therapy for 40 weeks: 15 mg qd, 30 mg QD, 45 mg QD	135 (efficacy cohort: 93)	CR at week 24 48% (22/46)	-	40% (37/93)	12% (11/93)
Upadacitinib	Induction therapy for 12 weeks: 45 mg QD	156	77.8% (121/156)	19.4% (30/156)	11.5% (18/156)	0.6% (1/156)

CR: Clinical Release; MH: Mucosal Healing; QD: Once daily.

## Abbreviations

*JAK inhibitor:* Janus kinase inhibitor; *IBD:* Inflammatory bowel disease; *STAT:* Signal transducer and activator of transcription; *CD:* Crohn’s disease; *UC:* Ulcerative colitis; *NOD2:* Nucleotide-binding oligomerization domain 2; *ATG16L1:* Autophagy-associated 16-like structural domain 1; *DCs:* Dendritic cells; *NLRs:* NOD-like receptors; *TLRs:* Toll-like receptors; *SCFAs:* Short-chain fatty acids; *TMAO:* Trimethylamine-N-oxide; *MDP:* Muramyl dipeptide; *RIPK2:* Receptor interacting protein kinase 2; *MAPK:* Mitogen-activated protein kinase; *MAVS:* Mitochondrial antiviral-signaling protein; *PGN:* Peptidoglycan; *OVA:* Ovalbumin; *IRF4:* Interferon regulatory factor 4; *IEC:* Intestinal epithelial cells; *CDH1:* Cadherin 1 gene; *HNF4A:* Hepatocyte nuclear factor 4 gene; *LAMB1:* Laminin subunit beta 1 gene; *ILCs:* Innate lymphocytes; *Th:* T helper cell; *Treg:* Regulatory T cell; *Tfh:* Follicular helper T cell; *PAMPs:* Pathogen-associated molecular patterns; *PRRs:* Pattern recognition receptors; *SMDs*: Small molecule drugs; *S1P:* Sphingosine-1-phosphate; *FGFR:* Fibroblast growth factor receptor; *VEGFR:* Vascular endothelial growth factor receptor; *PDGFR:* Platelet-derived growth factor receptor; *MMPs:* Matrix metalloproteinases; *RA:* Rheumatoid arthritis; *MACE:* Major adverse cardiovascular events; *VTE:* Venous thromboembolism; *AE:* Adverse event; *PY:* Person-year; *HZ:* herpes zoster; *DVT:* Deep vein thrombosis; *PE:* Pulmonary embolism; *RCT:* Randomized Controlled Trial; *QD:* Once daily; *BID:* Twice daily; *CR:* Clinical Release; *MH:* Mucosal Healing.
